# Vehicle-to-Vehicle (V2V) Message Content Plausibility Check for Platoons through Low-Power Beaconing †

**DOI:** 10.3390/s19245493

**Published:** 2019-12-12

**Authors:** Hyogon Kim, Taeho Kim

**Affiliations:** 1 Department of Computer Science and Engineering, Korea University, Anam-Dong, Sungbuk-Gu, Seoul 02841, Korea; 2 Department of Computer Science, University of Colorado Boulder, 1111 Engineering Drive ECOT 717, 430 UCB, Boulder, CO 80309-0430, USA; taeho.kim@colorado.edu

**Keywords:** V2V communication, message contents plausibility, power control

## Abstract

Although the IEEE Wireless Access in Vehicular Environment (WAVE) and 3GPP Cellular V2X deployments are imminent, their standards do not yet cover an important security aspect; the message content plausibility check. In safety-critical driving situations, vehicles cannot blindly trust the content of received safety messages, because an attacker may have forged false values in it in order to cause unsafe response from the receiving vehicles. In particular, the attacks mounted from remote, well-hidden positions around roads are considered the most apparent danger. So far, there have been three approaches to validating V2X message content: checking based on sensor fusion, behavior analysis, and communication constraints. This paper discusses the three existing approaches. In addition, it discusses a communication-based checking scheme that supplements the existing approaches. It uses low-power transmission of vehicle identifiers to identify remote attackers. We demonstrate its potential address in the case of an autonomous vehicle platooning application.

## 1. Introduction

In vehicle-to-everything (V2X) communications, various threats exist. They range from physical types such as signal jamming and Global Positioning System (GPS) spoofing to more logical types such as higher level protocol attacks or cryptanalyses. Unless thoroughly addressed, they can pose grave safety problems for human lives, not to mention less serious losses. Unfortunately, there is not a single overarching solution for all, due to the aforementioned diversity of the potential attacks. Each threat may require a different set of countermeasures.

The most notable security feature of V2X communication is the use of security credentials, a.k.a. certificates, commonly assumed by the Wireless Access in Vehicular Environment (WAVE) in IEEE 1609.2 [1] and the 3G Partnership Project (3GPP) Cellular V2X framework in 3GPP TR 36.885 [2]. Every safety message, the Society of Automotive Engineers (SAE) J2735 basic safety message (BSM) [3] broadcast from each vehicle in particular, is required to carry the certificate or its digest to provide the message source authentication and integrity check [1]. Also, it is used to provide privacy, by requiring frequent and continuous change [4].

However, one obvious threat that the certificate-based V2X security framework is not addressing is the message content fabrication. Unless appropriately filtered, vehicles can utilize received BSMs without knowing whether the contents of the messages sent by the nearby vehicles are genuine or fake. In case an attacker has access to the device with a valid credential, she could forge an erroneous message and transmit it, which will not be filtered by the certificate check. Then, the attacker could cause the receiver vehicles to inappropriately react to the faulty message content and possibly face critical consequences such as collisions. Since tampering with the sensory input data such as GPS position information to be transferred by communication protocols is easier than breaking the protocols or the cryptographic system, this type of attack may be more beneficial for the attackers.

Although the content fabrication attacks from a close distance can easily be refuted by the multitude of on-board sensors such as radars, cameras, and lidars on the vehicle, remote attacks out of the line of sight (LoS) of these sensors cannot be adequately filtered. A notable case would be roadside attacks, where the attacker can broadcast false information to the passing vehicles. For example, the attacker can fabricate false positional messages of a “ghost vehicle” to cause a multi-car pile-up in a long platoon [5]. Therefore, to obtain the users’ confidence in this technology, the credibility of the V2X messages from remote locations out of the range of the LoS sensors should be checked.

Below, we propose a solution where vehicles employ low-power beaconing messages (called “whispers”) to judge whether the vehicles can trust the contents of the received vehicle messages or not. This paper particularly focuses on the data fabrication attacks on the position information, a crucial and endangered subpart of the system [6]. We demonstrate the value of the proposed approach by considering a promising use case of V2X, platooning, so that the platoons can maintain traffic efficiency in the situation that the roadside attacker transmits fake messages. Although studies applying vehicular communication to truck platooning have dealt with these issues theoretically and practically Ref [7,8], the fact that platooning vehicles can receive vehicle messages containing false contents was not addressed in them. As the most effective attack target is the platoons, which is considered one of the promising applications of cooperative autonomous driving [9]. Finally, our proposal can be implemented only by utilizing the existing power control functionality that is already used for the congestion control in the V2X communication.

The contributions of this paper can be summarized as follows.

- By narrowing the spatial attack window to a small distance to the potential victims on the road, the proposed scheme can force the attackers into the detection range of the line-of-sight (LoS) hardware sensors. Thus, the scheme creates an opportunity for the cooperation between the LoS sensors and the communication for the message contents validation.
- By applying the proposed scheme to platooning, potentially the most vulnerable application of V2X to the false position attack, we show that it can mitigate not only the collision risk but also the discomfort from the unnecessary responses to the false position attack.

## 2. Related Work

The problem of message forgery has been noticed from the early days of V2X communication [10], to have dire safety consequences [11]. It is even considered the most serious security threat for its ease of implementation and execution [6]. Although the certifying authority (CA) is supposed to revoke the certificates on misbehaving devices as per the IEEE 1609.2 [1], there is an issue of how the authority can obtain and accumulate sufficient evidence that the devices are faulty or compromised [12]. Even if it could, it will take time during which the attacker has a time window of attacks to exploit. Especially if the attacker misbehaves in a discontinuous manner, namely evading detection intelligently, the task of identifying the misbehaving device and revoking its certificates will not be easy in the first place. So,  the message correctness check is considered the primary focus of misbehavior detection [13].

There have been mainly three approaches to solving the problem, i.e., behavior analysis [11,14,15], sensor-fusion [16,17,18] and communication-based constraint check [5,12,19]. These approaches are not mutually exclusive, so they can be used alone, or in combination. The first exploits physical constraints of vehicle movement dynamics. The second uses multiple sensor inputs to verify the position information in the received message. The third uses maximum communication range constraints. Below, we will briefly discuss these three that check the plausibility of the position information conveyed in a vehicle-to-vehicle (V2V) message. We note that because many of the existing approaches have been around for a long time, they do not reflect the recent standards development. It is one of the reasons that we need a new solution approach that is based on the standardized framework for the V2X communication.

### 2.1. Vehicle Dynamics-Based Validation

This approach relies on models of plausibility for vehicle behaviors. Laws of physics or driver behavior model can be used to judge if a claimed movement of a suspected vehicle is plausible. Stübing et al. [11] used Kalman filter to analyze the path taken by neighbor vehicles. By comparing the Kalman prediction of the path of a neighbor vehicle with the mobility data claimed in its periodic safety messages such as position, speed, and heading, this method judged if the deviation of the reported mobility data from the Kalman prediction is in an acceptable range; otherwise, it raises an alert. Finding that the Kalman filter cannot keep up with highly dynamic maneuvers such as sudden overtaking and hard braking, though, it additionally employed a hidden Markov model (HMM) to verify such movements. Sun et al. [20] also used Kalman filter on the received signal from the transmitter. Yavvari et al. [14] more extensively utilized the information contained in the BSM [3] to check the plausibility of the claimed movement (location and kinematics) by the message sender. Since the BSM has the lateral and longitudinal acceleration, speed, position, heading angle, and vehicle length and width, these data can be used to check the plausibility of the claimed movement given the past few transmissions from the message sender. If a claimed value exceeds the error range of the given dynamics model of the vehicle, the checking algorithm flags anomaly. Leinmüller et al. [19] tried to check if the V2X message has false position information by the fact that vehicles can move only at a well-defined maximum speed such as the general speed limit on streets. It also exploited the fact that only a restricted number of vehicles can reside in a certain area, whereby it can prevent Sybil attacks. It also used maps to check if a vehicle can navigate through the claimed position. Ghaleb et al. [15] used neural networks to find misbehavior in the communicated information. The local dynamic map (LDM) is constructed from the shared information, and each message is determined legitimate or malicious based on the historical behavior of the model.

### 2.2. Sensor-Based Cross-Checking

Bißmeyer et al. [16] used sensor fusion and particle filters to check the plausibility of the position information in the incoming messages and assign a trust level to the message sending vehicle. A separate particle filter was used for each tracked neighbor vehicle. The particle filter combines all available different position information from a variety of input sources such as V2X message, radar, road map, etc., to detect inconsistencies among them, introduced by faulty nodes or malicious attackers. For example, a radar, lidar, or camera could detect no neighbor vehicle at a position claimed through V2X. Not only each V2X message is evaluated in terms of the trustworthiness using the particle filter framework, but also the trust level of the message sending vehicle is computed based on the message trust rating. Schmidt et al. [17] presented a similar heuristic framework to combine the plausibility ratings from different sensor modules such as radars, lidars, and ultrasonic to check the message plausibility. The sensor-based check is part of a battery of tests using the movement analysis, sensor-proofed position check, minimum distance moved check, map-proofed position check, etc. Kim et al. [18] also combined various information sources such as sensors, maps, and input from other vehicles. Yan et al. [19] checked the position information in the received message through radar sensors. Each vehicle first monitors the neighboring vehicles using the radar in its range. Then it propagates the information to other vehicles to share the information globally. LeBlanc et al. [21] relies on road-side units (RSUs) to provide GPS reference values to defeat false position attacks.

### 2.3. Communication-Based Validation

The last approach to evaluate the trustworthiness of V2X reported position information uses the message conveying technology itself. First of all, a vehicle could compute the angle [22] or distance to the message sender using the received signal strength, the time-of-flight, and the angle-of-arrival of the V2X message. However, highly dynamic V2X channel conditions such as shadowing by blocking vehicles or multipaths created by metal-hulled vehicles make it hard to obtain reliable measurements. Moreover, the strict time synchronization between vehicles that is required for the computation would be hard to satisfy on the on-board units (OBUs) as these devices are not created for such purpose. Therefore, in this section, we will discuss only the schemes that rely on the most conservative constraints, i.e., the maximum possible communication distances.

Parno et al. [5] proposed a scheme where a vehicle’s relative location is defined by its entanglement with other vehicles. Each vehicle regularly broadcasts its identity (a public key) along with its signature of a current timestamp. When a vehicle *A* receives such a broadcast from another vehicle *B*, it signs *B*’s ID and rebroadcasts it. If a vehicle *C* on the opposite lane rebroadcast *A*’s identity before it rebroadcasts *B*’s identity, then *B* can conclude that *A* is ahead of him/her. As the authors noted, however, this scheme was a sketch of a possible solution and had strong assumptions to make it work. For one, it depends on the opposite-side traffic, a condition we cannot always rely on. However, the idea of entanglement within the communication range is viable, and we borrow it for our own proposal discussed later. Raya et al. [12] proposed a method to evict a misbehaving vehicle from the trusted set of neighbors even before the certifying authority (CA) includes the attacker’s certificates in the revocation list. In particular, if the attacker reports an implausible position that stands out from the observation of the honest majority, i.e., if messages are received beyond their expected area of propagation, the honest neighbors in the communication range of the attacker notice it. Then the honest majority begins to warn any new vehicle that comes into the communication range of the attacker to watch out for the potentially fabricated information from the suspected vehicle. Furthermore, if the accusation in the warning message collects enough supporting signatures from the honest majority, it promotes to the disregard message. When the disregard message with enough signatures is picked up by the roadside unit (RSU), it is forwarded to the CA so that it can revoke the certificates of the attacker. Leinmüller et al. [23] also exploited the maximum communication range limitation in addition to other behavioral anomalies of the vehicle in question to verify neighbors’ position information. In case an observing vehicle *M* overhears communication between *N* and *A*, it compares their positions and check if a possible attacker *A* can be within the maximum communication range of *N*. If the previously claimed position of *A* contradicts the condition, *M* considers *A* sending out false position information when it is actually at a position closer to *N*. Since this work is for multi-hop forwarding situation where the identity of the next forwarding node is known, we cannot directly apply it to the one-hop broadcast situation. Moreover, this checking method cannot be used against the remote attacker pretending to be close when it is actually far away.

Schmidt et al. [17] used a checking method that requires the neighboring vehicle should be heard during at least twice the maximum communication range $d_{TX}$, which is called the minimum distance moved (MDM), in order to be trusted. This check is to cope with stationary attackers whose transmission range is $d_{TX}$. Unfortunately, however, the current standards typically allow the adaptation of the transmit (Tx) power for the purpose of congestion control. In particular, the SAE J2945/1 stipulates that a Wireless Access in Vehicular Environment (WAVE) device can transmit at a Tx power ranging from 10 dBm to 23 dBm, depending on the channel congestion condition [24]. Consequently, a factor of 20 power difference obviously wildly affects the communication range of BSM. Moreover, the range can be also highly dependent on the given scenario and buildings in the vicinity, and be anywhere between 100 m and 500 m [13]. Under these circumstances, the position plausibility check based on the estimated maximum communication distance constraint cannot be reliable. Received signal strength indicator (RSSI)-based plausibility check mechanisms are subject to the same problem. Ruj et al. [25] exploits the time of flight to check the distance to the transmitter. But this scheme requires extremely precise time synchronization between vehicles.

Lastly, we stress that our paper is not about Sybil attacks. In Sybil attacks, the attacker can forge many false identities [26,27]. In the current V2X standards, however, the vehicle identity in each message must be proven by the attached security credential based on public key infrastructrure (PKI) Ref [1]. Our solution is based on the current standards, so we assume that even the attack should use its certificate to mount the attack. In our threat model, the attacker is not capable of tampering with the PKI. It can only tamper with the sensor input (e.g., Global Positioning System (GPS) coordinates) that is provided to the IEEE 1609.2 security module. In fact, it is the focus of this paper. When an authenticated attacker tries to propagate false information, we can narrow the spatial attack window to a short distance to the potential victims so that the hardware sensors can double-check the received false information.

In Section 3, we will introduce a complementary scheme in the third category, based on physical constraints of low-power communication, to defend against remote roadside attackers. Although it does not preclude the possible employment of the other two approaches in combination, an independent check based on the communication constraints has certain advantages over them. First, it can work even when the sensor-based position check does not work, e.g., the in non-line-of-sight (NLoS) condition. This is because most vehicle sensors such as camera, radar, and lidar are LoS devices. Second, it can work when the behavioral analysis may fail. For one, the behavioral analysis cannot be applied to a stopped vehicle, since it does not exhibit any movement behavior. Since V2X is expected to become a regulation-enforced safety feature in the near future [28], we believe the communication-based position verification should be included irrespective of other additional checks. In particular, our scheme can be easily implemented in the WAVE or the cellular V2X (C-V2X) framework.

## 3. Neighbor Verification through Low-Power Beacons

The core idea of our proposal builds on the physical communication constraint that messages from neighbor vehicles transmitted at a small transmit (Tx) power reach only the immediate proximity of the sender [29]. It contrasts with existing communication-based approaches that exploit the maximum communication range (e.g., 300 m) of the attacker as the constraint to be checked against [6,17]. In this section, we discuss our proposal to use low-power beaconing for proximity proving purpose, in order to defend against position data forging attack from stationary roadside attackers. The proposed scheme has a few desirable properties. First, it does not require a new hardware component beyond existing wireless access in vehicular environment (WAVE) on-board unit (OBU). Second, it does not excessively increase channel utilization as to hamper normal beaconing activities using BSMs [3]. Third, it works where sensor fusion is not applicable, and against a stationary attacker to which the behavior-based checking is not applicable. Below, we sketch the solution approach.

### 3.1. Sketch of Solution Approach

**Figure 1:**
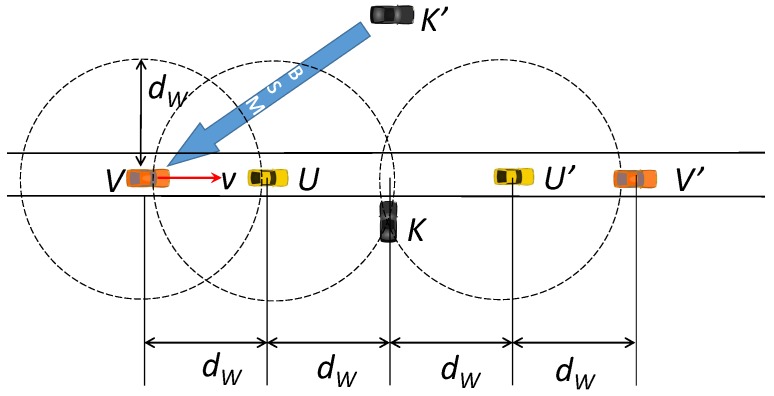
Low-power beacon checking setup.

See Figure 1 that depicts the movements of two honest vehicles *U* and *V* on a road strip, and two attackers *K* and $K^{\prime }$ on the roadsides. An attacker $K^{\prime }$ is less than $d_{B}$ apart from a potential victim *V*, where $d_{B}$ is the maximum distance that a BSM beacon can reach. Note that the attacker can use the standard Tx power (e.g., 23 dBm) or increase $d_{B}$ by using an amplifier or directional transmission. Without the proposed low-power beacon check scheme, $K^{\prime }$ can inject false positions or other safety-critical information in its BSM, $BSM_{K^{\prime }}$, and coax *V* into believing it and elicit a dangerous and unnecessary reaction (e.g., hard braking). But with the proposed low-power beaconing, each vehicle is designed to trust only those vehicles that are within the low-power beacon range $d_{W}\ll d_{B}$. It checks the condition not by the location information in neighbors’ BSMs, but per the physical constraints of wireless communication. Notice that it does not require the addition of new hardware or modification of the standard WAVE OBU, because the message Tx power can be explicitly controlled by application in WAVE short message protocol (WSMP) [30].

In our solution approach, each vehicle broadcasts special beacons carrying the sender’s randomly chosen identifier at much lower Tx power, in addition to BSM beacons. For convenience, we will call this special beacon “whisper” in the rest of this paper. Only if a neighbor echoes the received whisper identifier in its own whispers, the neighbor is trusted. In Figure 1, the remote attacker $K^{\prime }$ does not hear these whispers of *V*’s, as it is not within $d_{W}$ from *V*. Thus $K^{\prime }$ cannot include *V*’s identifier in its whispers, so *V* rejects the BSMs from $K^{\prime }$ as suspicious. For an attacker to mount the false position attack, therefore, it must come within the distance $d_{W}$ to the roadside. Moreover, it should exchange whispers with a passing vehicle (e.g., *U*) to enable an attack. For example, the attacker *K* in Figure 1 has a chance to mount the attack. Even in this case, the window of attack is limited to ±2$d_{W}$ in principle. So, with a sufficiently small $d_{W}$, we can drastically reduce the attack window for *K*. Furthermore, we can introduce an extension to this baseline scheme to further limit the attackers that come within $d_{W}$ from the roadside, which we will discuss in Section 3.6. Recollect that the purpose of limiting the Tx power of the whispers is to let only the neighbor vehicles in close proximity, but not a remote attacker, hear them. Finally, a desirable fallout from using a small Tx power for whispers is that it helps suppress the increase of the channel utilization due to this additional security measure to a small value. Even with this added security mechanism, therefore, we can still keep a larger chunk of the channel bandwidth for ordinary BSMs. Below, we discuss the details of the sketched idea.

### 3.2. Attacker Model

Before delving into the details of the proposed scheme, we specify the adversary model as follows.

- The attacker is stationary and located on the roadside [6,11], which will be the most frequent attack scene. The stationary attacker is identified in Leinmüller et al. [6] as the most threatening attack for its low complexity of implementation and execution.
- Before transmission, the attacker can tweak the data obtained from sensors or from the in-vehicle networks such as the controller area network (CAN) bus.
- The attacker has a valid security credential [11]. So, the attacker can correctly encode invalid position data.
- The attacker can increase the Tx power or use directional transmission to affect farther vehicles.

Note that the neighbor check using whispers is focused on the safety-related events that take place in the local neighborhood of each vehicle. To be precise, the local neighborhood is the two-hop range of the whispers (=2$d_{W}$). Thus the check completely prevents the attackers from placing “ghost vehicles” unless the attacker comes into the two-hop distance of the whispers. Then for the attackers that are indeed within the two-hop distance, we can develop an extension of the whisper scheme to further remove the attacks. For most safety-critical events, the attack prevention within 2 $d_{W}$ will be sufficient, as they take place in close proximity, e.g., forward collision. There will be safety applications where vehicles need to heed messages from long distances, but they are beyond the scope of this paper.

### 3.3. Neighbor Check through Low-Power Beaconing

In the WAVE framework, every vehicle periodically transmits a beacon called BSM, at up to 10 Hz. Such safety beaconing is similarly performed in C-V2X as well, with two more higher rates (20 Hz and 50 Hz) [31]. In this paper, we additionally require that every vehicle *V* transmit special beacons called whispers, denoted by $W_{V}$, that carry the following information:

- $I_{V}$: whisper identifier (WID) of *V*
- $L_{V}=\left\{I_{x}|x\in N_{V}^{t}\right\}$: list of WIDs heard by *V*, where $N_{V}^{t}$ is one-hop neighbors that passed the neighborhood check using whispers
- $dig(C_{V})$: digest of *V*’s certificate [1]

The whisper identifier (WID) $I_{V}$ is randomly chosen by each vehicle, and changed every update interval $t_{u}$, much more frequently than the pseudonym change [24]. (the WID should also change upon the pseudonym replacement as stipulated by SAE J2945 [24], as well as every $t_{u}$.) Otherwise, once the attacker learns of $I_{V}$, it would be able to prove itself as a close neighbor of *V* by using $I_{V}$. Then the attacker could attack *V* with false information as long as its forged BSM can reach *V*. By changing $I_{V}$ frequently, however, the vehicle *V* can make it hard for the attackers that once learned of $I_{V}$ to attack *V* later in time.

Every vehicle *V* puts in $L_{V}$ all whispered neighbor identifiers $I_{x}$ that heard and passed its “whisper check”, and rebroadcasts them in its own whisper $W_{V}$. The whisper check refers to the following test: if a receiver *U* of $W_{V}$ finds its whisper identifier $I_{U}\in L_{V}$, *U* trusts the content in subsequent beacons $BSM_{V}$ to come from a close neighbor *V*. If a neighbor vehicle fails the whisper check, on the other hand, its whisper identifier (WID) is neither stored nor rebroadcast. The one-hop whisper neighborhood $N_{V}$ of *V* is defined by the maximum distance from which a whisper reaches *V*. $N_{V}^{t}\subseteq N_{V}$ is the subset of the one-hop neighborhood that passed the whisper check.

Each vehicle *V* stores the binding $\left(I_{x},\;dig\left(C_{x}\right)\right)$ for each neighbor $x\in N_{V}^{t}$. This is to prevent impersonation attacks. Suppose *V* has received a whisper from *U* and confirmed $I_{V}\in L_{U}$. At this moment, the binding $\left(I_{U},\;dig\left(C_{U}\right)\right)$ is made at *V*. Once the binding is made, *K* cannot impersonate *U* because it does not have the private key of *U* to sign the BSM that is validated only by $C_{U}$. On the other hand, suppose that *K* hears the whisper and the BSM from *U* before the binding $\left(I_{U},\;dig\left(C_{U}\right)\right)$ is made at *V*. In this case, *K* can attempt to pretend to be *U* and make a biding $\left(I_{U},\;dig\left(C_{K}\right)\right)$ at *V*. However, *U* is not (yet) a trusted neighbor of *V*, so the whisper $W_{U}$ does not have $I_{V}$. Therefore, *K* could not prove that it is a one-hop whisper neighbor of *V*. When $BSM_{K}$ reaches *V*, *V* will reject this forged BSM.

Algorithm 1 shows the description of the proposed whisper checking logic. Line 3 is the whisper check. If the received whisper fails this test, the sender is determined to be unverifiable as a neighbor (line 16). If the whisper check succeeds, the whisper ID is used as a key to find the bound certificate (line 4). If none, we create one to prevent the impersonation attack (line 5). If there is a binding, however, the WID-certificate binding is checked against the values in the received whisper (line 9). If they match, it is a confirmation of both the neighbor relation and the WID-certificate mapping. The trust is strengthened as the credit for the received whisper is incremented (line 10). This information will be later used in an enhanced version of the base algorithm discussed here. If the binding and the whisper say otherwise, the whisper is ignored (line 12). Note that the binding should be newly created when either the pseudonym change or WID change takes place.
**Algorithm 1** Whisper check at *U*.1:**procedure**Whisper-Check($W_{V}$)              ▹*U* received whisper $W_{V}$ from *V*2:    Extract $L_{V}$, $I_{V}$, and $dig(C_{V})$ from $W_{V}$3:    **if** $I_{U}\in L_{V}$ **then**                       ▹ Did *V* hear my whisper?4:        **if** $B_{U}\left(I_{V}\right)==\emptyset$ **then**  ▹*B* is the WID-certificate binding set; No binding exists for *V* yet5:           $B_{U}\left(I_{V}\right)\leftarrow dig\left(C_{V}\right)$          ▹ Make one for *V*; $C_{V}$ is the link to BSM from *V*6:           $L_{U}\leftarrow L_{U}\cup I_{V}$             ▹ Store *V*’s whisper ID (WID) for rebroadcast7:           8:        **else**                             ▹ Binding exists for $I_{V}$9:           **if** $B_{U}\left(I_{V}\right)==dig\left(C_{V}\right)$ **then**                 ▹ Binding confirmed?10:               $C(I_{V})$++                               ▹ Credit up11:           **else**                                   ▹ Conflict12:               return                              ▹ Ignore this whisper13:           **end if**14:        **end if**15:    **else**16:        return                       ▹ Ignore this unverifiable neighbor17:    **end if**18:**end procedure**

### 3.4. Range Extension

One obvious question will be about the appropriate value of the whisper range $d_{W}$. It should depend on applications that require contents plausibility checks. What if an application requires a wider checking range? One may argue that we could increase the Tx power of whispers to extend the range, but it would increase the channel utilization as well. As a consequence, it can trigger the BSM congestion control [24] so that fewer BSMs are transmitted and the vehicle position tracking error increases. In essence, it can have safety ramifications. In order to extend the range of whisper check to two hops without such side effects, we could take an approach similar to Parno et al. [5]. Namely, when $W_{V}$ passes the whisper check at *U*, we can let *U* not only trust BSMs from *V*, but also those from the vehicles in $L_{V}$. So *U* can identify two-hop trusted neighbors $N_{U}^{t^{2}}=⋃L_{x}$, where $x\in N_{U}^{t}$.

**Figure 2:**
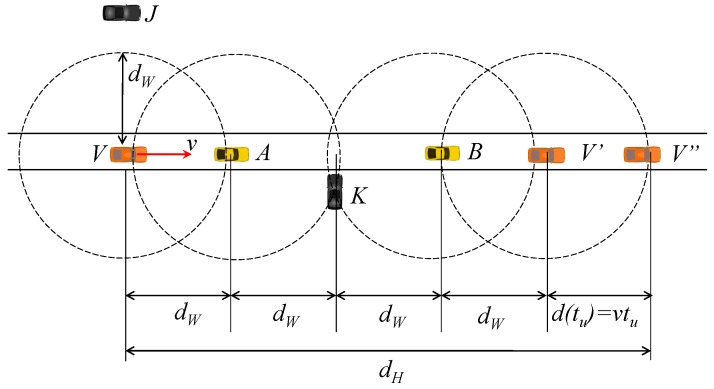
Distances in credit-based whisper.

Unfortunately, under the two-hop whisper check, when the distance $d_{K}$ between the attacker’s true position and the message receiving vehicles is less than $d_{W}$, the attacker can also exploit the overheard whisper identifiers (WIDs) to extend the attack range. Figure 2 depicts the situation. Here, the attacker *K* can obtain $I_{V}$ from $L_{A}$, for $A\in N_{V}^{t}$ and $d\left(A,\;K\right)\leq d_{W}$. Echoing this overheard $I_{V}$ in its whisper $W_{K}$, the attacker can extend its attack range from $d_{W}$ to $2d_{W}$. Not only that, after *V* passes *K*, a similar situation can happen if there is another intermediate vehicle (e.g., *B*) between the attacker and the victim.

Furthermore, there are two more factors that can extend the attacker’s reach even beyond $2d_{W}$. The first is the whisper ID update. Recollect that the WID is changed every $t_{u}$ (see Section 3.3). If vehicle *V* moves at speed *v*, it can add as much as $vt_{u}$ to $d_{K}$ because *V* can continue to use the same $I_{v}$ even after moving more than 2 $d_{W}$ away from *K*, before the next update instant. The second factor is the message gap between consecutive whispers. Suppose *V* changes its WID from $I_{v}$ to $I_{v}^{\prime }$. Since the update will be reflected in the next whisper to transmit, a neighbor *U* can send its BSM containing the old WID $I_{v}$. If *V* performs the whisper check against its new WID $I_{v}^{\prime }$, this legitimate BSM from *U* will be filtered. Therefore, before whispering with the new WID, each vehicle should accept BSMs with its old WID. Assuming the whispering rate of *c* Hz, neighbors BSM with the old WID can arrive as late as 1/c second after the update, which we reflect to the attacker’s overhearing range calculation. In total, the attacker can attempt to deceive a vehicle at distances

(1) $$d_{X}\leq 4\cdot d_{W}+v\cdot \left(t_{u}+1/c\right),,$$

where $d_{X}$ is the maximum distance that the attacker can exploit *V*’s WID. We stress that the extended attack range $d_{X}$ is not where the attacker can mount the attack from. The attacker should still be physically within $d_{W}$ of the road to overhear the whispers. In this paper, however, we shun away from the multi-hop whispering possibilities due to the potential to extend the attacker’s capability. We leave it as future work and focus on exploring the efficacy of the single-hop whispering.

Note that the changed mobility model can affect the attack distance $d_{X}$. In particular, the increased vehicle speed *v* affects Equation (1), extending the attack distance. In this case, according to the same equation, more rapidly changing the whisper ID (namely, smaller $t_{u}$) can be used to counter it.

### 3.5. Whispering Rate and Tx Power

**Figure 3:**
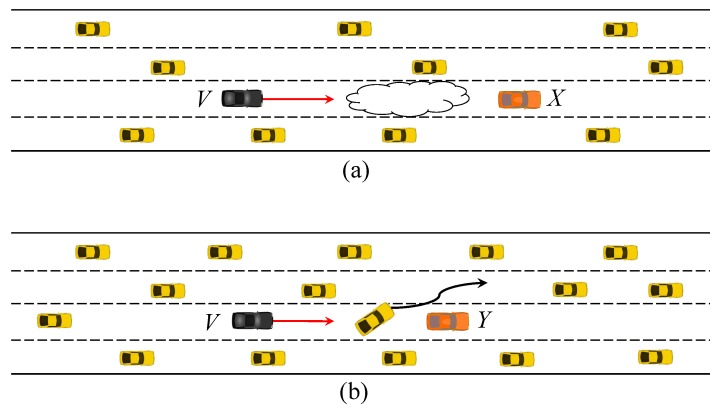
Highway driving simulation scenarios (**a**) poor visibility (**b**) blocked line of sight.

Lowering the Tx power of whispers helps filter the messages from remote roadside attackers and reduce the channel utilization incurred by the whispers. But the Tx power set too low can render the BMS from even close neighbors not trusted. Therefore, we should find an appropriate Tx power. As to the channel utilization, another parameter that affects it is the whispering rate. To find appropriate whispering Tx power and frequency to be used for the rest of this paper, let us consider two highway driving scenarios depicted in Figure 3. There are four lanes on the simulated road. The BSM Tx power is set to 23 dBm, and the messaging rate to 10 Hz. On the other hand, the Tx power of whispers is varied between 7 and 10 dBm, and the messaging rate between 6 and 8 Hz. The channel model is set to two-ray ground. The physical layer transmission rate is set to 6 Mbps for the most robust delivery [32].

**Figure 4:**
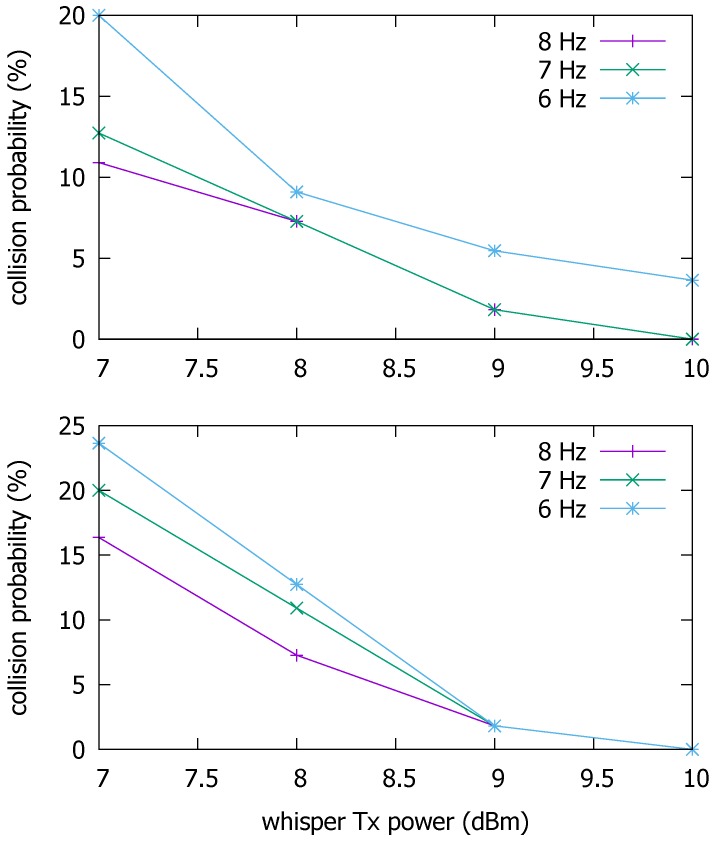
Vehicle collision probabilities as functions of messaging rate and Tx power.

In (a), vehicles move at 80 km/h in the rainy road condition, where the road surface friction coefficient is 0.3 [33]. The headway distance between vehicles on the same lane is set to 100 m. In this case, the host vehicle *V* cannot detect the stopped vehicle *X* due to poor visibility. In (b), vehicles move at 120 km/h on dry road where the friction coefficient is 0.8. The headway distance between vehicles on the same lane is set to 50 m. There is a vehicle between *V* and the stopped vehicle *X*. The stopped vehicle warns approaching vehicles through BSMs. Other vehicles check the distance to the stationary vehicle at the moment of receiving the first BSM that passes the whisper check. In (b), the intervening vehicle changes lanes to evade *Y*. The human reaction time typically ranges from 0.7 s to 1.5 s [34]. In this paper, we set it to 1 s. When vehicles move at 80 km/h on a rainy road or 120 km/h on a dry road, the braking distances based on our assumptions are 104.5 m and 102.8 m, respectively. Given these parameters, we compute the distance *d* between the host vehicle and the stopped vehicle when a whisper checked the first BSM from the stopped vehicle arrives at the host vehicle. Based on *d*, Figure 4 shows the probabilities in the two scenarios that the host vehicle collides with the stopped vehicle as it could not stop in time. The collision depends on the message delivery loss ratio and latency experienced by the BSM from the stopped vehicle. Now, given the success probability of the forged message delivery, the vehicle collision takes place if

$$d+v_{f}\cdot \frac{v}{\left|a\right|}<v\cdot \left(1+\frac{v}{\left|a\right|}\right)+\frac{v^{2}}{2a},$$

where *d* is the headway distance to the front vehicle *U*, $v_{f}$ is the speed of *U*, *v* is the speed of the host vehicle *V*, *a* is the maximum deceleration.

Let $d_{ca}$ denote the distance between the BSM sender and the receiver at which the collision probability is less than 5%. In (a), the message reception is possible at distances $d>d_{ca}$ at 9 dBm and 7 or 8 Hz. In (b), all three messaging rates qualify at 9 dBm. For this reason, we will assume in the rest of this paper that the whisper Tx power is set to 9 dBm, and the whispering frequency to 7 Hz. Note that these numbers are only used as rough guidelines to demonstrate the potential of the proposed approach. If need be, they will be refined in future work.

### 3.6. Further Check Based on Credit

**Figure 5:**
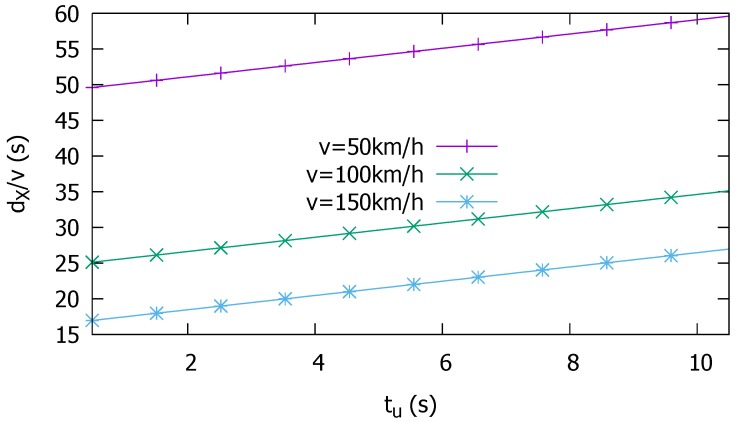
Elapsed time required for trust vs. whisper time allowed for the attacker, $d_{W}=170$ m.

An attacker within $d_{W}$ of the roadside can still hear the whispers of the target vehicles and mount the attack. In order to cope with such attackers, we can extend the whisper scheme by incorporating the notion of credit. In this extension, each vehicle *V* tracks the credit $C_{V}\left(U\right)$ for each neighbor vehicle *U*. Without further sophistication, we simply let the credit increase by one point per each passed whisper test and decrease by one point each second. In Algorithm 1, we showed how the credit for each neighbor is increased. To prevent the attacker from arbitrarily inflating the credit, the increase for each neighbor can be bounded by the whispering frequency $f_{W}$, so that the reception of whispers beyond $f_{W}$ does not add to the credit. Assuming the minimum whisper rate cannot fall below 1 Hz, legitimate neighbors within $d_{W}$ of *V* will maintain a non-zero credit at *V*. Given the whispering frequency of $f_{W}=7$ Hz, for instance, the maximum credit that one neighbor can accumulate per second is 7 − 1 = 6. Based on this observation, we can set the credit threshold over which we can trust a given neighbor vehicle at *V* as

(2) $$\theta_{V}=\left(f_{W}-1\right)\cdot d_{X}/v\left(V\right),,$$

where v(·) is the speed of the given vehicle. The threshold θ is essentially the credit that a roadside attacker can maximally accumulate at *V* while *V* travels a distance $d_{X}$ at *v*, or equivalently, during $d_{X}/v\left(V\right)$ seconds. Here, $d_{X}$ is the maximum distance that a vehicle can use the same whisper ID, as in Equation (1). To show how long a neighbor vehicle should travel with a host vehicle to be considered credible, Figure 5 plots $d_{X}/v\left(V\right)$ as a function of $t_{u}$ and *v* for $d_{W}=170$ m.

A single-hop whisper check will roughly halve θ. Therefore, in reality, the time that neighbor vehicles should be in the communication range of a host vehicle to be trusted can be small. The credit-based enhancement ascertains $C_{V}\left(U\right)>\theta_{V}$ for *V* to trust the message from *U*. Namely, if the neighboring vehicle *U* can accumulate more than a roadside attacker maximally can, *V* trusts *U*.

Obviously, there is a risk of completely ignoring the legitimate vehicles whose credit falls short of θ, e.g., due to message losses arising from adverse channel conditions. Or, a new vehicle can join the traffic from a junction. But Figure 5 is a very conservative estimate considering that we define $d_{W}$ to be the maximum distance at which the whisper reception probability is non-zero. The whisper ID can change much faster, as long as the binding with the certificate digest is maintained. Using small $t_{u}$ reduces θ, helping vehicles within the whisper range $d_{W}$ quickly exceed the threshold for each other. Using smaller $d_{W}$ would reduce the threshold even further, especially when *v* is small, reducing $d_{W}$ by using smaller Tx powers can be useful because the smaller distance is driven in a given time than in high-speed driving so that credibility checking may be more focused on closer neighborhood.

## 4. Attack Filtering Performance

**Figure 6:**
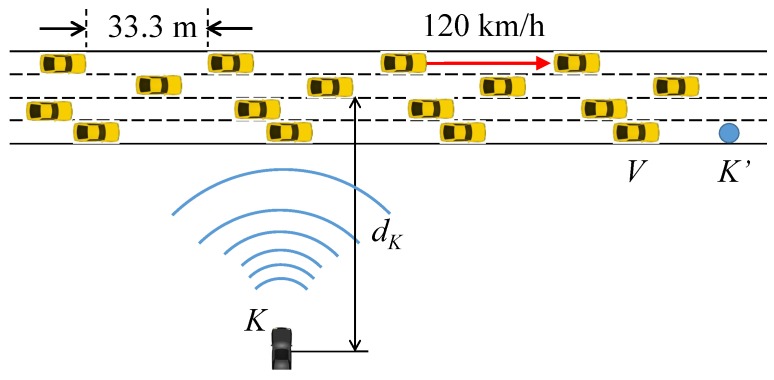
Attack scenario.

In this section, we investigate the attack filtering performance of the proposed scheme, by simulating a highway driving scenario. Figure 6 depicts the attack situation used in simulation. Vehicles are moving on a 4-lane highway at the same speed of 120 km/h. The headway distance between vehicles on the same lane is 33.3 m, or equivalently, a 1 s gap at the given speed. The whisper size is the sum of the whisper ID (2 bytes), the digest of the certificate (8 bytes) [1], and the list of received whisper IDs (at most 90 × 2 bytes). The BSM size 80 bytes for the message and 125 bytes for the certificate, so it is 201 bytes. The whisper and BSM are transmitted as a WSMP message in IEEE 802.11 frame, and the lower layer overhead is an additional 80 bytes. In this paper, we assume that BSMs are transmitted at 10 Hz, and whispers at 7 Hz. Note that at such whispering rate, legitimate vehicles that fail to deliver some of the whispers due to the poor channel condition will succeed within seconds at most, and assert their neighborship without being suspected for long. The power of BSMs is 23 dBm, and whispers, 9 dBm. The path loss model is two-ray ground, and the fading model is Rician with k=3. We perform the simulation using the Qualnet simulator, and the simulation configuration is summarized in Table 1. In the given situation, we consider the attack successful at a vehicle *V* if *V* hears $BSM_{K}$ with a false position information (i.e., that of $K^{\prime }\neq K$) and $BSM_{K}$ passes *V*’s whisper test. Then, we count through simulation the number of vehicles that are successfully attacked for varying distances of attacker to the road, $d_{K}$.

### 4.1. Attack Mitigation Performance

**Figure 7:**
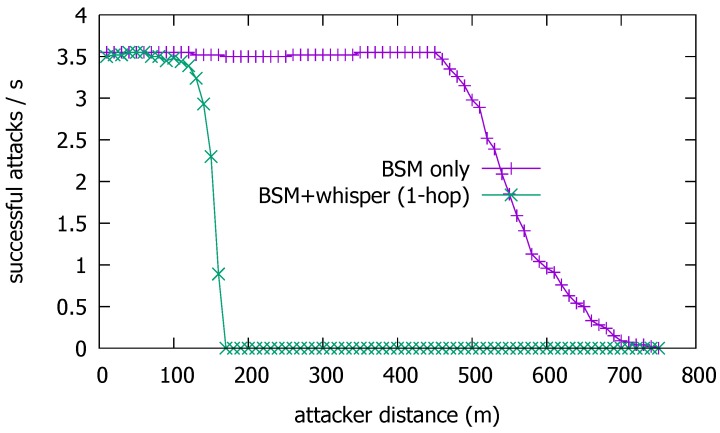
Successful attacks with basic safety message (BSM) only and with BSM + whisper.

As discussed above, if $d_{K}>d_{W}$, then $I_{v}\in L_{K}$, so *V* can filter *K*’s BSM. This holds true even if *K* increases the BSM transmission power or uses directional transmission to extend its transmission range towards farther victim vehicles. But as *V* comes closer so that its whisper can be decoded by *K*, then *K*’s BSM can pass *V*’s test. Figure 7 shows the number of successful attacks as a function of $d_{K}$. Namely, it plots how far from the road center the attacker can successfully mount the attack. Although the number of vehicles that fall to the attack is also a function of the vehicle traffic density, vehicle speed, and the duration of the attack, we fix them as in the previous section and focus on the effect of $d_{K}$. Without the whisper check, the attacker can successfully achieve the attack from as far as $d_{K}>600$ m at the BSM Tx power of 23 dBm. With Tx power-boosting or directional transmission, $d_{K}$ could be even larger. This result suggests that with good line-of-sight (LoS), the attacker may well position himself safely apart from the highway, avoid visual detection by the passing victims, and still pose a significant threat. The reason that the number of vehicles exposed to the attack is approximately 3.5 inside the attack range is because we have a headway distance of 33.3 m between vehicles. The length of a vehicle is 5 m, so with four lanes, approximately 3.5 vehicles enter the attack range every second. With the whisper check, however, the attack enabling distance $d_{K}$ is significantly reduced to $d_{W}\approx 170$ m. The result confirms that the whisper check is effective in narrowing the attack range to $d_{W}$. So, at least, the attacker should come near to the roadside in order to mount the false position attack under the whisper scheme.

### 4.2. Channel Utilization Increase

**Figure 8:**
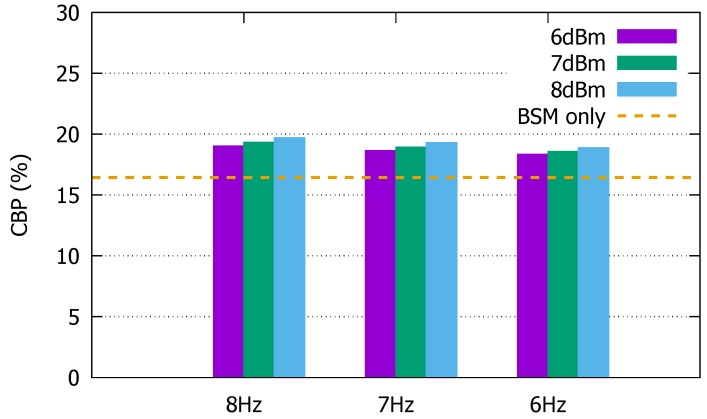
Channel busy percentage (CBP) increase due to whispers.

Additional whispering activity inevitably increases channel utilization. If excessive, it could even jeopardize the more important safety message exchanges such as BSM. So, we also measure the channel used in the simulation to check on this possibility. Figure 8 shows the channel busy percentage (CBP) as a result of using whispers. We notice that the increase in CBP remains at 2% to 3% in each of the considered variations of Tx power and frequency. Even if the whisper messaging rate is 60% to 80% of the BSM beaconing rate, the increase in CBP is not as significant due to the smaller power at which the whisper messages are transmitted. So, the low-power beaconing scheme only slightly increases the CBP, and it can be done without excessively disturbing the ordinary beacon exchanges.

### 4.3. Effectiveness of Credit-Based Enhancement

**Figure 9:**
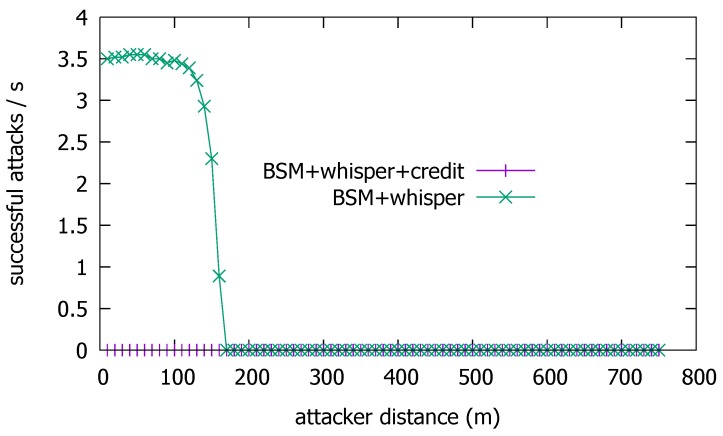
Attack success probability with additional credit-based check.

To check the efficacy of the credit-based enhancement, we repeat the simulation for Figure 6 with the employment of the credit-based check. Figure 9 shows the result.

We see from the figure that the additional credit-based check completely shuts out the road-side attacker, as it fails to accumulate enough credit, just as intended by Equation (2). A crucial observation in the issue of position plausibility check is that the risk of vehicle collisions is higher between those that are in close proximity. Therefore, it is imperative that the nearby vehicle positions should be ascertained more than those of farther vehicles. It is why the SAE J2945/1 standard stipulates that only the vehicles within 100 m of the ego vehicle be tracked [24]. From this viewpoint, the credit-based double check is highly recommended due to its usefulness in checking the positions within short, safety-critical distances, $d_{W}$ in particular.

We can summarize the significance of the proposed scheme as follows. In connected/autonomous cars, V2X is no longer an option, but a mandatory component that allows vehicles to sense larger distances and non-line-of-sight (NLoS) situations. Without the proposed solution, the attackers can safely position themselves at a significantly larger distance from the victims (connected/autonomous cars). The hardware sensors such as cameras, radars, and lidar are all line-of-sight (LoS) sensors, and their coverage is limited. Therefore, with only the sensors, we could not cope with the attacks that use longer range and non-LoS technology that is the V2X. By using a smaller Tx power to make $d_{W}$ narrow, we can create an opportunity for the cooperation between the LoS sensors and the communication for the message contents validation.

## 5. Application to Platoon Protection

As traffic and cargo volume increase worldwide, many researchers have studied how to reduce traffic congestion and to carry cargo efficiently through truck platooning. In 2011, California PATH [35] team conducted experiments with three heavy trucks that have only an automated longitudinal control and confirmed the improvement of fuel consumption by about 10% on the average. Energy ITS [36] team performed tests with three automated heavy trucks and one light truck with the gaps of 10 m and 4.7 m at 80 km/h in 2013. Currently, there are numerous others seeking even higher efficiency goals [37]. These project teams conducted their research using vehicle sensors and vehicle-to-vehicle (V2V) communication, and they demonstrated that platooning can improve energy [38] and traffic efficiency [39]. Like this, as platooning is one of the most promising applications of V2X [9], and one of the prominent target for roadside remote attackers when V2X becomes available [5], it is imperative to explore how the proposed scheme can help mitigate attacks against platoons.

### 5.1. Problem Formulation

**Figure 10:**
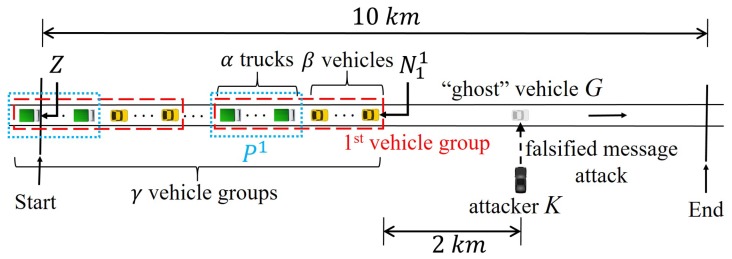
Simulated platooning scenario.

In platoons, each vehicle can utilize V2V communication to obtain information about vehicles that are in non-line-of-sight (NLoS) points that cannot be detected by its sensors. This is also essential for all platooning vehicles to use the platoon leader’s driving information. For our purpose, it is considerably difficult to define the platoon formation and the surrounding traffic in a completely generic manner. Thus in this paper, we consider the scenario as depicted in Figure 10, where the autonomous platooning and non-platooning vehicles move together on a road, and they are subjected to a roadside attack from *K*.

Since the platooning is dangerous to perform in urban roads, the highway environment is usually assumed. Figure 10 is the platooning scenario in the highway environment. Following the 3GPP TR 36.885 (Annex A) suggests for evaluation scenarios [2], we used a straight road longer than 2 km. Unlike the 36.885 that suggests 2.5 s gap between vehicles that run at 70 km/h, however, all non-platoon vehicles in our paper try to keep at 80 km/h with 1 s gap (approximately 22 m) and platoon vehicles, 10 m.

In this formulation, there are γ vehicle groups on a 10 km stretch of a lane. Each vehicle group is composed of α+β vehicles, where α and β are the numbers of platooning vehicles and non-platooning vehicles, respectively. It models the driving situation from the viewpoint of platoons where it is behind a non-platoon vehicle population in a lane. As the platoon uses a smaller inter-vehicle gap than non-platoon vehicles, the false position attack from *K* is a more grave safety threat to the platoon vehicles. As above, all vehicles exchange BSM at 23 dBm, but for whispers, we will assume here that they use the Tx power of 9 dBm, slightly higher than in the previous sections. As a result, $d_{W}$ is 170 m in our simulation setting while BSM propagation dwindles in power till 600 m beyond which the packet delivery is hardly possible.

Let $N_{j}^{i}$ be the $i^{\mathrm{th}}$ non-platooning vehicle of the $j^{\mathrm{th}}$ group, and $P^{k}$ denote the *k*th platoon. For the string stability of the platoons, we model into the simulator the Rajamani controller [40] that computes the desired acceleration of each vehicle in the platoon. For this, we assume that the platoon vehicles receive the movement data of both the preceding vehicle and the platoon leader through V2V communication, whereas non-platoon vehicles receive only those of the preceding vehicle. We set the intra-platoon safety gap, namely the required minimum distance between two consecutive vehicles in the platoon, to 10 m as assumed in many works of literature. The length of a vehicle is 5 m, and the safety gap between non-platooning vehicles or between a non-platooning vehicle and the immediately following platoon leader is one second headway time at 80 km/h, which is approximately 22 m. Although presenting the case where collisions occur will obviously be more dramatic, we set the safety gap to a large value because we want to show that even if there is no collision, the attack can still affect the comfort and the efficiency of the vehicle traffic running with platoons.

We assume that all platoon vehicles try to keep their speed at 80 km/h. In contrast, non-platoon vehicles can use higher speeds when it wants to close the gap with a preceding vehicle. The maximum acceleration and deceleration of both types of vehicles are assumed to be 3 m/s${}^{2}$ and −5 m/s${}^{2}$, respectively [41]. All vehicles can use their sensors to detect the obstacles within 150 m of the line-of-sight (LoS). Notice that $N_{1}^{1}$ has no obstruction in front, so it can easily recognize obstacles in the front. However, the front view of its followers are blocked by the preceding vehicles, making it difficult to detect obstacles. An attacker *K* is situated at $d_{W}$ from the roadside. Although a very remote attacker can be more easily rejected as explained above, by putting the attacker within the hearing range of the whisper, we want to stress-test the proposed scheme. *K* broadcasts forged messages announcing a ghost vehicle *G*, which is not actually present, at a distance of 2 km ahead of $N_{1}^{1}$. *K* continuously broadcasts forged messages, causing the vehicles to brake when the vehicle groups come in the range of its BSM transmission.

### 5.2. Effect of Whisper Check with Platoons

We first measure the time it takes for the last vehicle *Z* of the entire vehicle groups to pass through the endpoint of the 10 km stretch, while varying α, β and γ(5≤α≤10,1≤β,γ≤10). It will reveal any accumulated delay effect if the vehicles in front are affected by the forged message attacks. Specifically, without the whisper check, each vehicle with a block front view will believe that *G* is actually present at the forged coordinate. If the vehicle determines that there is a risk of colliding with *G*, it will activate its brake. For this, we assume that each vehicle brakes at 30% of the maximum deceleration when the time to collision (TTC) with the preceding vehicle is 2.6 s, and at the maximum deceleration when the TTC is 1 s to prevent collision [42]. Each vehicle activates the brake until its LoS sensors finally perceive that *G* is not actually present.

**Figure 11:**
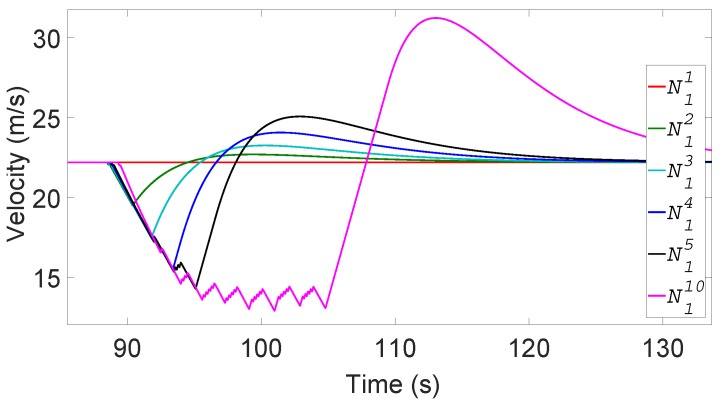
Velocity changes of the non-platoon vehicles in the first vehicle group without forged message filtering.

When the whisper check is not employed, Figure 11 shows the velocity changes of some of the non-platoon vehicles in the first vehicle group under the forged message attack that a ghost vehicle is at *G*. We assume α=β=10 in this example.

Since $N_{1}^{1}$ has the LoS for the ghost vehicle *G*, it can use its sensors to perceive that *K*’s messages are forged and keeps going at the same velocity as before without braking. But as for the other vehicles in the group, they brake to slow down due to the absence of LoS to *G*. So, $N_{1}^{2}$ begins to brake at 30% of the maximum braking when its TTC to *G* reaches 2.6 s. But as soon as $N_{1}^{1}$ passes the forged position, the LoS to the forged coordinate is clear for $N_{1}^{2}$. Its sensors detect that *G* does not exist, so accelerates again to reach the target safe distance with $N_{1}^{1}$ (at around t=90 s in Figure 11). Notice that the velocity of the following vehicles more severely decreases towards the end of the non-platoon vehicle column. Due to the slowed vehicles in front, the latter vehicles spend more time until they reach *G*, when their sensors find the absence of the ghost vehicle. Some middle vehicles are not shown for readability, but this accumulated slowdown effect is amplified to a very large and elongated velocity instability as it propagates to the last non-platoon vehicle $N_{1}^{10}$. From the velocity changes of $N_{1}^{10}$, we observe in the simulation data that its acceleration and deceleration occurs as many as 60 times, potentially causing discomfort to the passengers.

**Figure 12:**
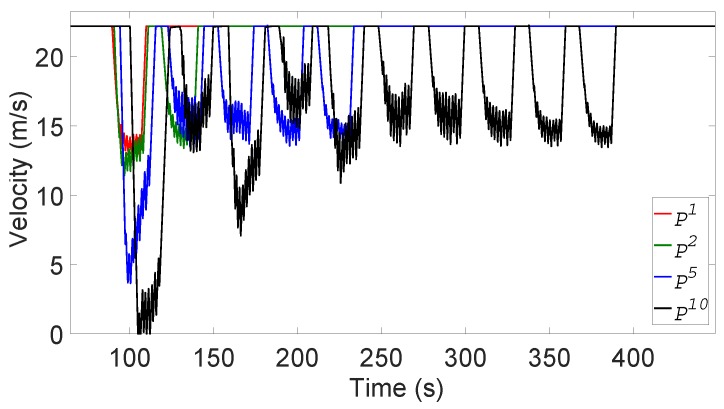
Velocity changes of platoons without forged message filtering.

The velocity fluctuation in the non-platoon vehicles directly affects the immediately following platoon vehicles. The leader of the platoon $P^{1}$ that immediately follows $N_{1}^{10}$ has to slow down due to the attack and then increases its speed to the original speed only after its other sensors check the absence of *G*. However, unlike the non-platoon vehicles, the platoon members of $P^{1}$ are controlled by the leader, so at the command of the leader, they move at the same speed. Recollect that the platoon members are prohibited from accelerating to more than 80 km/h. The other platoons $P^{2},\ldots ,P^{10}$ experience similar dynamics. Furthermore, the speed of $P^{i}$ decreases more than that of $P^{i-1}$ as the effect accumulates. Figure 12 shows the velocity changes of some of the platoons. In general, $P^{i}$ suffers *i* episodes of significant velocity fluctuation due to the remote attack. We observe that the latter platoons, most notably $P^{10}$, suffer from elongated and repeated instability.

**Figure 13:**
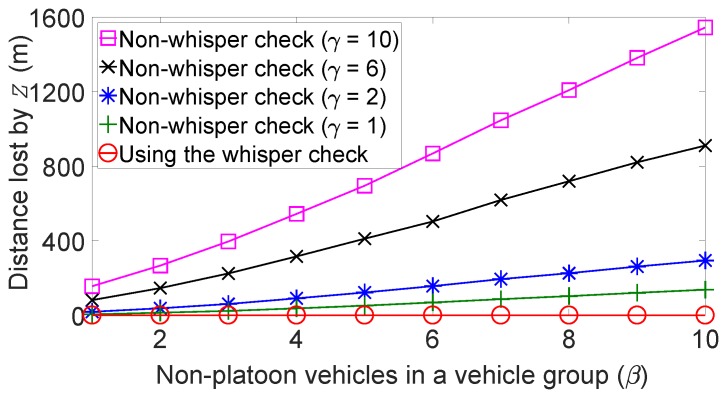
Velocity changes of platoons without forged message filtering.

Using the whisper check can drastically reduce the undesirable impacts of the remote forged position attack such as exposed by Figure 11 and Figure 12. Figure 13 sheds light on these impacts of whisper checks from yet another angle, the distance loss. In particular, it shows the distance lost by the last vehicle *Z* compared with the case in which vehicles employ the whisper checks, under various values of β and γ and α=10. We fix the value of α because the platoon followers travel at the same speed as the platoon leader and had little effect on the velocity change of *Z*. When all vehicles use the whisper check, *Z* travels 10 km at a velocity of 80 km/h without any distance loss, regardless of β and γ values, because the vehicles successfully filtered out forged messages. In contrast, in the absence of the whisper check, the average velocity of *Z* decreases as β and γ increase, and there is lost distance. For instance, for α=β=γ=10, the average velocity of *Z* while moving 10 km dropped to about 52 km/h, far below 80 km/h with the whisper check in play.

Although we set the inter-vehicle distance to sufficiently large values so that the roadside attack did not cause direct collisions, the loss of average speed and the potential discomfort due to repeated and wild fluctuations of the vehicle speed would be enough to annoy the vehicle riders. If the inter-vehicle distance were lower for higher road throughput or fuel-efficiency, it would increase the probability of more serious events.

## 6. Conclusions

In this paper, we explore a Tx power-based communication constraint check that can filter remote attacks that aims to disseminate false position information to running vehicles. By using low-power beacons, vehicles can mutually check if the BSM hence the position information therein indeed comes from a physically close neighbor. At least, it would pressure an attacker to come close within the low-power transmission range of the victim vehicles to mount an effective attack. In case the attacker indeed comes in close range, the on-board hardware sensors such as radars, lidar, and cameras with a typically smaller range than V2X can kick in to validate the claimed position.

Through extensive simulation, we demonstrated that there is value in using the low-power beacon exchanges between vehicles in preventing the harmful impacts from remote false position attacks through V2X communication. Specifically, we confirm that traffic efficiency and comfort of platooning may be decreased due to the remote attack. We show, however, that if we employ the low-power beaconing message check to platooning, we can successfully cope with forged message attacks and can overcome the problem. The additional bandwidth cost is small thanks to the low Tx power, and the Tx power reduction for the additional beacons is easily implementable within the current V2X standard frameworks.

## Figures and Tables

**Table 1:** Simulation parameters.

Parameter	Value	Explanation
$P_{W}$	9 dBm	Whisper Tx power
$f_{W}$	7 Hz	Whisper frequency
$L_{W}$	≤194 B	Whisper size
$f_{B}$	10 Hz	BSM frequency
$P_{B}$	23 dBm	BSM Tx power
$L_{B}$	205 B	BSM size
*v*	120 km/h	Vehicle speed
$d_{I}$	33.3 m	Headway distance
Path loss	Two-ray ground	
Fading	Rician (K=3)	
